# Of Mouse and Man: Cross-Species Characterization of Hypertensive Cardiac Remodeling

**DOI:** 10.3390/ijms23147709

**Published:** 2022-07-12

**Authors:** Susanna T. E. Cooper, Joseph D. Westaby, Zoe H. R. Haines, Giles O. Malone, Mary N. Sheppard, Daniel N. Meijles

**Affiliations:** 1 Molecular and Clinical Sciences Institute, St. George’s University of London, Blackshaw Rd., London SW17 0RE, UK; m1907428@sgul.ac.uk (S.T.E.C.); jwestaby@sgul.ac.uk (J.D.W.); p1807508@sgul.ac.uk (Z.H.R.H.); gilesmalone@icloud.com (G.O.M.); msheppar@sgul.ac.uk (M.N.S.); 2 CRY Cardiovascular Pathology Department, St. George’s Healthcare NHS Trust, St. George’s University of London, Blackshaw Rd., London SW17 0QT, UK

**Keywords:** hypertension, cardiac remodeling, quantification

## Abstract

Hypertension is a major public health concern and poses a significant risk for sudden cardiac death (SCD). However, the characterisation of human tissues tends to be macroscopic, with little appreciation for the quantification of the pathological remodelling responsible for the advancement of the disease. While the components of hypertensive remodelling are well established, the timeline and comparative quantification of pathological changes in hypertension have not been shown before. Here, we sought to identify the phasing of cardiac remodelling with hypertension using post-mortem tissue from SCD patients with early and advanced hypertensive heart disease (HHD). In order to study and quantify the progression of phenotypic changes, human specimens were contrasted to a well-described angiotensin-II-mediated hypertensive mouse model. While cardiomyocyte hypertrophy is an early adaptive response in the mouse that stabilises in established hypertension and declines as the disease progresses, this finding did not translate to the human setting. In contrast, optimising fibrosis quantification methods and applying them to each setting identified perivascular fibrosis as the prevailing possible cause for overall disease progression. Indeed, assessing myocardial inflammation highlights CD45+ inflammatory cell infiltration that precedes fibrosis and is an early-phase event in response to elevated arterial pressures that may underscore perivascular remodelling. Along with aetiology insight, we highlight cross-species comparison for quantification of cardiac remodelling in human hypertension. As such, this platform could assist with the development of therapies specific to the disease phase rather than targeting global components of hypertension, such as blood pressure lowering.

## 1. Introduction

Hypertension is colloquially and simply defined as abnormally high blood pressure; however, the molecular causation and remodeling responses are far more complex. While it is known that hypertension promotes left ventricular hypertrophy [1] and fibrosis [2], the intricacies of the progression to a diseased state in humans remain unspecified. As the advancement of hypertension can result in cardiac failure and sudden cardiac death (SCD), attributed to hypertensive heart disease (HHD) [3], elucidating the various phases in disease progression is essential for future therapeutic design, notably as blood pressure lowering alone fails to halt disease progression [4].

The use of a mouse model to study hypertension and the associated cardiac maladaptation is well published [5]. However, to the best of our knowledge, direct extrapolation of murine responses has never been directly applied to, or correlated with, human disease. Mechanistically, studies in genetic mice [6] or wider rodent models [7] have identified specific phases of hypertensive remodeling. Generally, the primary phase involves increasing cardiomyocyte size (hypertrophy) as an adaptation to increased arterial pressures [8]. In contrast, maladaptive responses occur in a secondary phase where excess fibrotic deposit stiffens the heart resulting in the clinical presentation of diastolic dysfunction [8]. However, underscoring each of these phases are additional processes that contribute to the changing myocardial architecture. For example, the “inflammation hypothesis” is re-emerging as a potential driver of heart disease progression in hypertension [9]. Specifically, myocardial inflammation involves the recruitment of inflammatory cells that cause paracrine or autocrine signaling between cardiac-centric cells that is associated with cardiomyocyte hypertrophy [10], cell injury [11] and fibroblast activation [12].

In this study, we sought to quantify features of human hypertensive cardiac remodeling using parameters defined from murine hypertension studies [13,14]. Specifically, we assessed cardiomyocyte hypertrophy, perivascular fibrosis and inflammatory cell infiltration in hypertensive mouse hearts to quantify modifications within the human heart as hypertension advances to HHD. By measuring hypertension-induced hypertrophic and fibrotic changes, along with inflammation and the infiltration of inflammatory cells, we provide a platform that can aid the development of novel targeted treatment specific to the disease phase. Moreover, we believe that this method advancement outlines inflammation as a key profile affecting myocardial remodeling that needs to be evaluated in all hypertension-linked studies, irrespective of species.

## 2. Results

### 2.1. Cardiomyocyte Hypertrophy Is an Acute Phase in Hypertension

**Figure 1:**
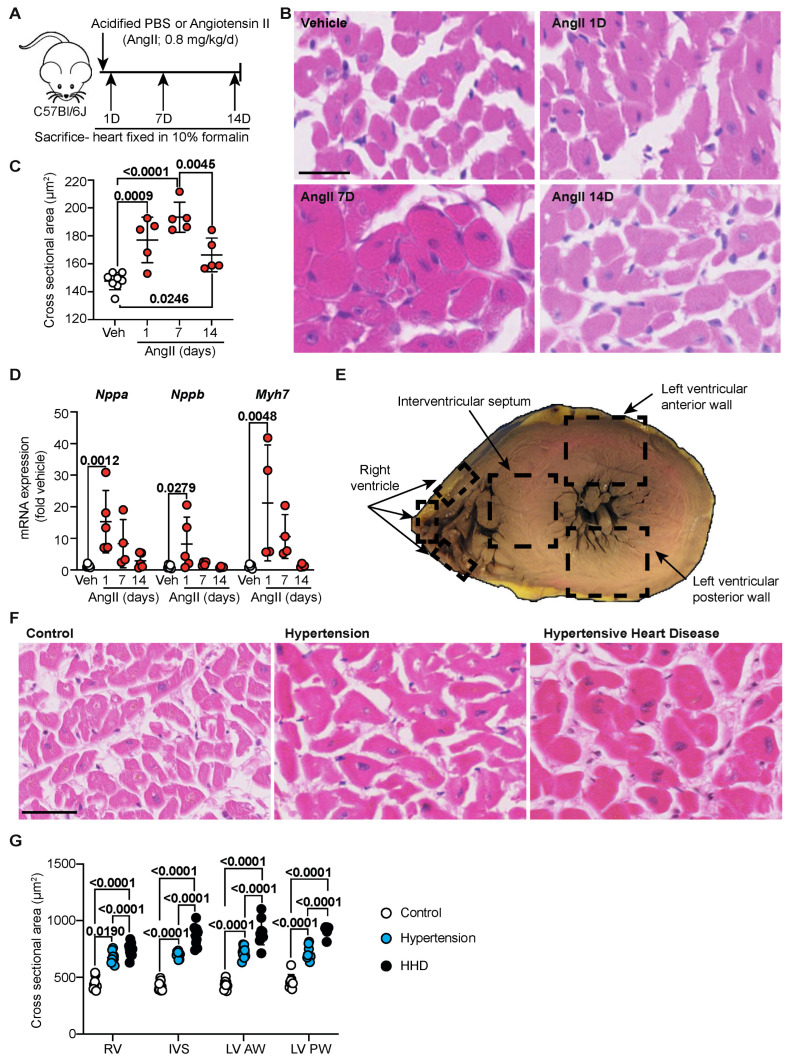
Cardiomyocyte hypertrophy is an acute phase in hypertension. (**A**) Schematic of hypertensive murine model. C57Bl/6J wild-type mice treated with either vehicle (Acidified PBS; *n* = 10) or Angiotensin II (AngII; 0.8 mg/kg/day) for 1 d, 7 d or 14 d (*n* = 4–5/group). (**B**) Representative images of murine heart sections stained with haematoxylin and eosin (H&E). Line represents 25 µm. (**C**) Quantification of murine cardiomyocyte cross-sectional area. Data are individual points with means ± SD. Stats: 1-way ANOVA with Holm–Sidak post-test. (**D**) Mouse cardiac RNA was isolated and mRNA expression of hypertrophy-associated genes determined by quantitative polymerase chain reaction. Data are individual points with means ± SD. Stats: 1-way ANOVA with Holm–Sidak post-test. (**E**) Schematic indicating regions of the human heart from which sections were taken for analysis. (**F**) Representative images of human left ventricular anterior wall stained with H&E. Line represents 50 µm. (**G**) Quantification of cross-sectional area of cardiomyocytes (control, *n* = 10; hypertension, *n* = 9; hypertensive heart disease, *n* = 10) across the whole human heart. Data are individual points with means ± SD. Stats: 1-way ANOVA with Holm–Sidak post-test. RV—right ventricle; IVS—interventricular septum; LV—left ventricle; AW—anterior wall; PW—posterior wall.

Hypertension is known to cause cardiomyocyte hypertrophy as a result of increased arterial pressure [15]. Being a primary hormone responsible for inducing hypertension in humans and a well-established model in mice, we sought to understand the adaptive response of the heart to angiotensin-II (AngII; Figure 1A) in mice over a 2-week period [13,14,16]. Compared to vehicle, AngII rapidly increases cardiomyocyte cross-sectional area within 1 day of infusion, a response that peaked by 7 days (Figure 1B,C). By 14 days, however, myocyte size reduced, indicating a potential switch in the disease phase to HHD. Indeed, analysis of pro-hypertrophic Nppa, Nppb and Myh7 mRNA markers supports this with a significant increase within 1 d of AngII infusion, which remained elevated to 7 day (Figure 1D) in line with our previous studies [14]. Significance, however, was masked by the heightened 1-day response and gradually declined to baseline by 14 days. We next investigated whether this observation extended to humans using post-mortem cardiac tissue collected from four areas across the heart: the right ventricle (RV), intraventricular septum (IVS) and left ventricular (LV) anterior and posterior walls (Figure 1E). Cardiomyocyte size in clinically hypertensive and HHD individuals was significantly increased compared to controls in all regions of the heart. It was notable in the clinically hypertensive group that, from the context of macroscopic/gross and microscopic examination, the heart was deemed normal (Figure 1F,G). In terms of HHD, however, a further increase in myocyte size was identified compared to clinical hypertension, which was present throughout all examined regions of the heart.

### 2.2. Perivascular Fibrosis Predominates in Human and Murine Hypertensive Remodelling

**Figure 2:**
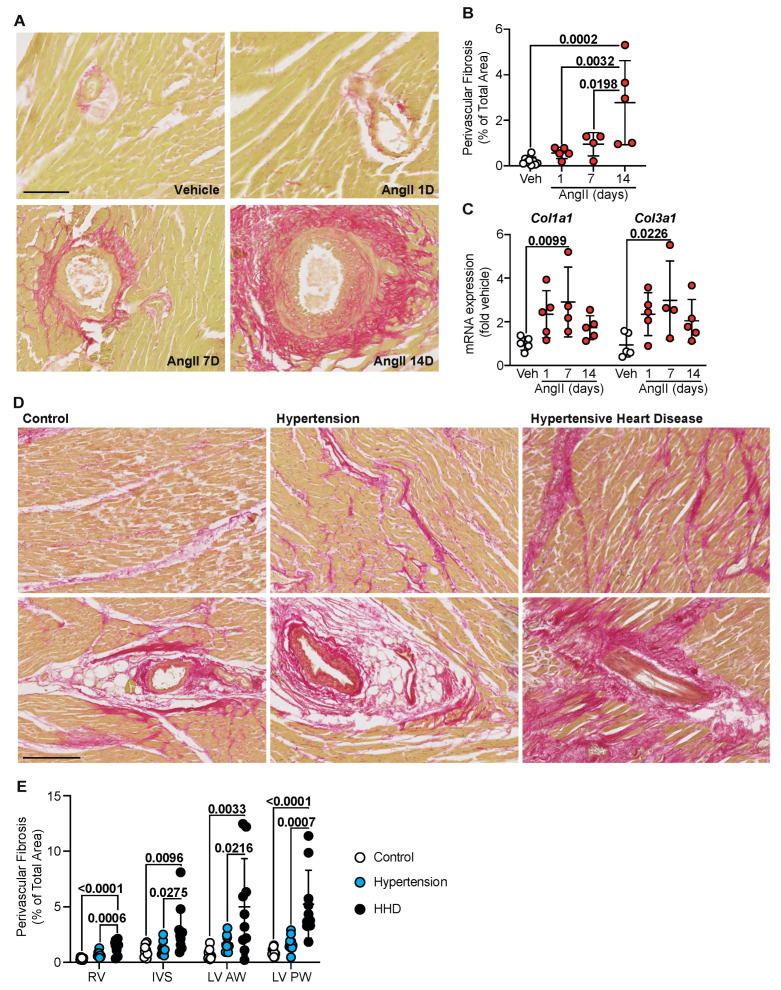
Perivascular fibrosis predominates in human and murine hypertensive heart disease. (**A**) C57Bl/6J wild-type mice treated with Angiotensin II (AngII; 0.8 mg/kg/day) for up to 14 days. Representative images of heart sections stained with picrosirius red (PSR). Line represents 100 µm. (**B**) Quantification of perivascular fibrosis as a percentage of total tissue area in the murine left ventricle. Data are individual points with means ± SD. Stats: 1-way ANOVA with Holm–Sidak post-test. (**C**) Ventricular RNA was isolated and mRNA expression of fibrillar pathological collagens determined by quantitative polymerase chain reaction. Data are individual points with means ± SD. Stats: 1-way ANOVA with Holm–Sidak post-test. (**D**) Representative images of human left ventricular anterior wall stained with PSR for interstitial fibrosis (top) and perivascular fibrosis (bottom). Line represents 250 µm. (**E**) Quantification of perivascular fibrosis as a percentage of total tissue area across the whole human heart. Data are individual points with means ± SD. Stats: 1-way ANOVA with Holm–Sidak post-test. RV—right ventricle; IVS—interventricular septum; LV—left ventricle; AW—anterior wall; PW—posterior wall.

AngII infusion has been found to induce cardiac fibrosis by causing fibroblasts to accumulate and deposit collagen [13,17]. We looked to assess the progression of fibrotic remodelling in the mouse model by staining left ventricular tissue for fibrillar collagens using picrosirius red (PSR). As expected, AngII infusion increased total fibrosis; however, this response was delayed, supporting our previous studies [13,14]. Interestingly, perivascular fibrosis became increasingly evident as hypertension and time progressed, with extensive fibrotic deposits observed around the vessels seen from 7 days (Figure 2A,B). Indeed, assessment of fibrillar collagen mRNAs supported this delayed fibrotic response where Col1a1 and Col3a1 showed a maximal increase at 7 days and were sustained to 14 days (Figure 2C). In humans, a slight but non-significant increase in PSR-labelled perivascular fibrosis was observed in the clinically hypertensive group across all regions of the heart assayed (Figure 2D bottom, Figure 2E). Upon progression to HHD, however, a marked increase in the perivascular fibrosis area was evident. Interstitial fibrosis, though not specifically quantified, was noted and predominantly appeared to be connected and spreading from the perivascular response (Figure 2D top). As such, perivascular fibrosis appears to be the dominant cardiac remodelling process associated with disease progression as it is seen at significant levels both in human HHD and in murine hypertension as a chronic-second phase response.

### 2.3. Inverse Relationship between Perivascular Fibrosis and Cardiomyocyte Hypertrophy in Advanced Hypertension

**Figure 3:**
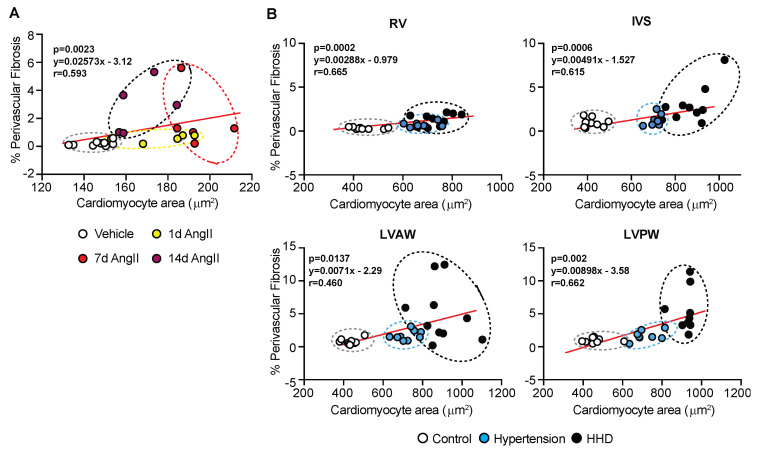
Cardiomyocyte size and perivascular fibrosis correlation with disease progression. (**A**) C57Bl/6J wild-type mice treated with Angiotensin II (AngII; 0.8 mg/kg/d) for up to 14 d. Correlation of cardiomyocyte cross-sectional area with perivascular fibrosis (as a percentage of total area). (**B**) Human post-mortem cardiac tissue with hypertension and hypertensive heart disease. Correlation of cardiomyocyte cross-sectional area with perivascular fibrosis (as a percentage of total area). Stats: Pearson correlation, with 2-tailed test reporting *p*-values.

AngII-hypertension in mice is bi-phasic, with an initial response that is cardiomyocyte driven and a secondary phase that appears to be fibrosis-dependent (Figure 1 and Figure 2). To establish connection between these remodelling components and extrapolate to the human scenario, we correlated cardiomyocyte size to perivascular fibrosis during disease progression. In the mouse AngII model, a linear correlation between LV-cardiomyocyte hypertrophy and perivascular fibrosis for up to 7 days was identified (Figure 3A). However, by 14 days, this relationship switched, likely reflecting disease progression. In humans, a similar correlation was evident, with myocyte size increasing along with perivascular fibrosis (Figure 3B). Interestingly, the LV-anterior wall scatter is atypical to other regions and more aligned with the extended timeframe of the murine model, suggesting that the region of the heart adapts ahead of other regions, possibly due to the associated vessel supply and other myocardial remodelling processes.

### 2.4. Inflammation Is a Catalyst for Hypertensive Cardiac Remodelling

**Figure 4:**
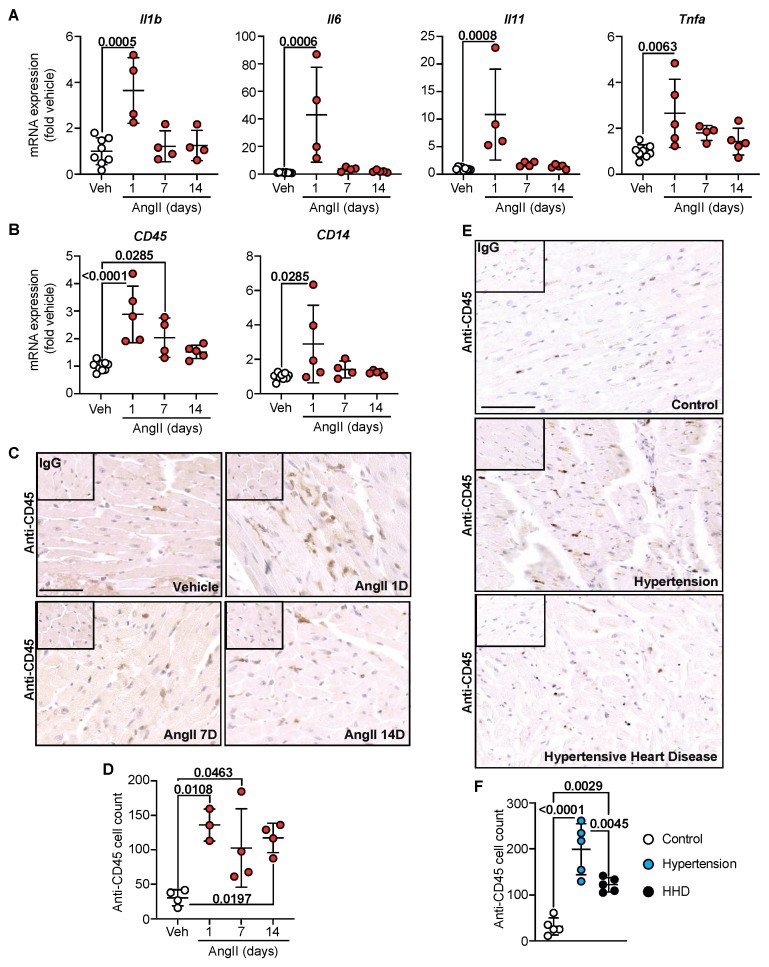
Inflammation initiates hypertensive cardiac remodelling. C57Bl/6J wild-type mice treated with Angiotensin II (AngII; 0.8 mg/kg/day) for up to 14 day. Cardiac RNA was isolated, and mRNA expression of pro-inflammatory cytokines (**A**) and general inflammatory cell markers (**B**) were determined by quantitative polymerase chain reaction. Data are individual points with means ± SD. Stats: 1-way ANOVA with Holm–Sidak post-test. (**C**) Representative images of murine left ventricle immunostained for CD45. Line represents 50 µm. Inset: IgG-negative controls. (**D**) Quantification of CD45+ cells in murine left ventricle. Data are individual points with means ± SD. Stats: 1-way ANOVA with Holm–Sidak post-test. (**E**) Representative images of human left ventricular anterior wall immunostained for CD45. Line represents 100 µm. Inset: IgG-negative controls. (**F**) Quantification of CD45+ cells in human left ventricular anterior wall. Data are individual points with means ± SD. Stats: 1-way ANOVA with Holm–Sidak post-test.

**Figure 5:**
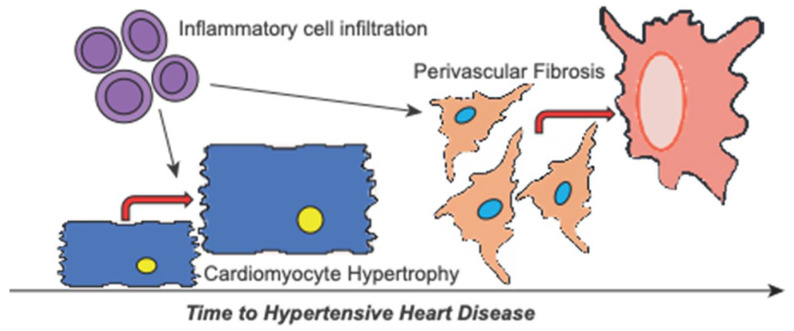
Schematic of cardiac remodelling involved in hypertensive heart disease progression. Hypertension causes cardiac remodelling with hypertensive heart disease marked by global cardiac hypertrophy and notable fibrosis. Our data indicate that remodelling is primed by increased pro-inflammatory signalling and inflammatory cell infiltration. Subsequently, inflammation encourages further hypertrophy of the cardiomyocytes contributing to the early remodelling phase. Over time, this promotes the progression of hypertensive heart disease towards failure resulting from increasing perivascular fibrosis.

Alongside hypertrophic and fibrotic responses, emerging evidence supports myocardial inflammation in the pathophysiology of heart failure [18]. Indeed, AngII in mice is a known inducer of sterile inflammation [13]. Whether myocardial inflammation precedes or is consequential to fibrotic remodelling is undefined. In hypertensive mouse hearts, mRNA expression of pro-inflammatory cytokines Il1b, Il6, Il11 and Tnfa was significantly elevated within 1 day of AngII infusion, a response that waned by 14 days (Figure 4A). These are known inducers of inflammatory cell infiltration. As such, mRNA expression of the general inflammatory cell marker CD45 and the activated monocyte/macrophage marker CD14 mirrored pro-inflammatory cytokine profiles, with significant elevations within 1 day of AngII treatment (Figure 4B). Moreover, confirmatory immunohistochemical labelling for myocardial CD45-positive inflammatory cells supported mRNA profiles, where a significant increase in immune cells was noted within 1 d of AngII treatment and remained elevated through to 14 days (Figure 4C,D). In the human cohort, a similar effect was observed: clinical hypertension was associated with the highest level of CD45-positive labelling (Figure 4E,F), and levels dropped as disease progressed to HHD. This study, therefore, suggests that inflammation may be a crucial trigger for adverse hypertensive cardiac remodelling that predisposes to perivascular fibrosis and correlates to the progression of hypertensive heart disease (Figure 5).

## 3. Discussion

Hypertension represents a significant cause of morbidity and mortality globally, with adverse cardiac remodeling predisposing to arrhythmias and leading to heart failure or sudden cardiac death. Our study sought to identify whether distinct phases of hypertensive cardiac remodeling exist and if these processes could influence hypertensive heart disease progression. Specifically, by applying quantification criteria from mice to humans, we show perivascular fibrosis is a key remodeling event that predisposes to disease progression in both species. Moreover, we demonstrate that cardiac inflammation is an early-phase response to hypertensive (e.g., AngII) stimuli in mice and is also linked to HHD progression in post-mortem characterized human hearts (summarized in Figure 5).

Hypertensive heart disease is characterized by left ventricular hypertrophy. Cardiomyocyte hypertrophy is the primary mechanism by which the heart adapts, aiming to reduce ventricular wall stress induced by pressure overload, such as in the setting of hypertension [15]. By optimizing murine quantification methods to the human setting, we were able to microscopically identify cell-based responses that would otherwise be missed upon standard post-mortem analysis. Indeed, this mode of assessment has been successful in several cross-species genome sequencing studies [19,20]; however, it has not before been applied to a quantitative species-comparison of remodeling. Our results in human hearts highlight that an early response to hypertension is a uniform increase in myocyte size to combat the rise in pressure, which precedes discernibly abnormal macroscopic wall dimension increases (Figure 1). Additionally of note is the increased myocyte size in the right ventricle, which is typically overlooked in the setting of hypertension, where the focus for diagnosis is left ventricular hypertrophy [1]. Our results, therefore, showcase the microscopic vs. macroscopic intricacies of the remodeling heart, which could be of assistance to pathologists in implicating hypertension when heart size is increased but death has occurred prior to any clinical signs noted, and there is no formal diagnosis of hypertension.

Extensive cardiac remodeling, whereby the accumulation of collagens type I and III results in cardiac fibrosis, plays a central role in the pathogenesis of hypertension [2,8]. Fibrosis is known as the substrate for arrhythmia and therefore indicates the susceptibility to SCD as the disease progresses [21]. The majority of literature discussing the etiology of hypertensive remodeling and targeted therapies only highlights the fibrotic response as interstitial, with the topic of vessels relating to the risk of atherosclerosis development [1,22]. With respect to fibrotic quantification, as opposed to techniques utilizing color threshold or colored pixel intensity, the assessment of the fibrotic area presented here allows for interstitial and perivascular fibrosis to be distinguished. We believe this to be fundamental to understanding the progression of the disease as clinical studies have indicated that perivascular fibrosis is independent of the interstitial response [23]. Perivascular fibrosis is a dysfunctional effect and is elevated in non-ischemic heart failure induced by a myriad of cardiac disease settings, including hypertensive heart disease (Figure 2), along with hypertrophic and dilated cardiomyopathies [23]. Furthermore, significant increases in perivascular fibrosis are noted in rats with hypertension-induced heart failure [24]. Our results indicate that global cardiac hypertrophy, deemed a marker of advanced hypertension [25], could be due to the build-up of fibrosis around the vessels as opposed to the interstitial response or increased myocyte size (Figure 2). Indeed, correlate analysis demonstrates how the disease progresses, where the anterior wall of the human left ventricle aligns with the murine response (Figure 3). This is expected based on the size and significance of the vessel supplying the anterior wall of the heart, namely the left anterior descending (LAD) coronary artery. Supporting studies, therefore, identify perivascular fibrosis in areas supplied by the LAD, which were significantly associated with coronary microvascular dysfunction [23]. Ultimately, a heart is going to fail when it is no longer able to provide essential supplies to the periphery, a response dependent on the LV. As our data indicate the implication of perivascular fibrosis in disease progression, the chances of progress to failure are thus higher in this region of the heart. Ultimately, highlighting the significance of fibrotic scar around the vessels could provide a new target for hypertensive treatment to prevent further progression towards advanced coronary vessel remodeling and overall organ failure.

It is well established that pro-inflammatory cells are pivotal in the development of cardiovascular disease [18]. For example, increased microvascular dysfunction correlates with peripheral fibrosis, typified by global inflammation and extravasation of immune cells into tissues [23]. Additionally, it is evident that immune cells respond to cardiac injury much earlier than was initially thought, with our data (Figure 4) confirming these changes prior to the maladaptive hypertrophy seen macroscopically with hypertension [10]. With respect to cardiomyocyte hypertrophy, several studies have suggested a role for pro-growth factors, such as endothelin-1 (ET-1), as key mediators of chronic inflammation, increasing tissue macrophages and lymphocyte infiltration [26]. While ET-1 is a potent vasoconstrictor, it also promotes hypertrophic remodeling of cardiomyocytes, and its formation and release are known to be stimulated by AngII [27,28]. It is, therefore, possible that myocyte hypertrophy in hypertension is mediated, in part, by ET-1 induction of the inflammatory response. Furthermore, in a diseased state, cytokines promote the differentiation of fibroblast to myofibroblast, depositing collagen initially in an attempt to repair cardiac damage [29]. However, the activation of fibroblasts releases further cytokines to target the ‘wound’, which in turn activates resident macrophages and can perpetuate the immune response [30]. For example, previous studies have indicated that the activation of macrophages and T cells in hypertensive hearts increases Monocyte Chemoattractant Protein 1 (MCP-1), which maintains immune cell recruitment, leading to sustained inflammation [18]. The cycle of immune response and fibroblast activation thus maintains fibroblast proliferation which can become detrimental if inadequately controlled, resulting in chronic inflammation and pathological fibrosis [31]. Here, we looked at pro-inflammatory cytokines, of which IL-11 has also been found to have a central role in the development of cardiac fibrosis [32], thus echoing the fundamental association between inflammation and fibrosis. Additionally, the inflammatory response can further increase blood pressure, driving the pathological remodeling seen within the heart as a result of hypertension [33]. Over time, this leads to cardiac stiffening and creates scarring through which electrical conduction is blocked (aka diastolic dysfunction), thereby increasing the chance of an arrhythmic event and SCD. As our data indicate increased inflammation in the early stages of hypertension, with extensive cardiac remodeling continuing with disease progression, the advancement of the disease could be attributed to early but sustained and self-renewing inflammation. Ultimately, we believe that the progression of the perivascular fibrotic response is because of inflammation, based on the extravasation of inflammatory cell infiltration into the cardiac tissue. Indeed, expert pathological assessment of CD45+ cells identified ~40% of CD45+ cells in control human tissue were macrophage-like, with a typical elongated appearance compared to round lymphocytes. Interestingly, these macrophage-like cells increased to ~50% with clinical hypertension and dropped to ~30% with HHD. In mice, however, ~20% of CD45+ cells appeared macrophage-like, with a jump to ~55% within 1 day of AngII infusion. The decrease in inflammatory cell infiltrate seen with disease progression could be a result of cell differentiation and loss of CD45 expression while still contributing to the inflammatory profile. As such, the initial inflammatory response could be a result of several different inflammatory cells in the acute phase, with a lower proportion of chronic inflammatory cells, such as lymphocytes or histiocytes, persisting with disease progression. A small body of literature now exists highlighting the role of inflammatory cell sub-types in hypertensive remodeling. However, there is still no consensus as to what the infiltration of inflammatory cells does to the heart following an inflammation-triggering event. For example, regulatory T cells have been found to be cardioprotective following myocardial infarction, whereas they are also linked with the advancement of myocarditis [34,35]. Furthermore, pressure overload hypertrophy by trans-aortic constriction causes cardiac resident macrophages to regulate positive myocardial adaptation, while non-resident infiltrating macrophages proved detrimental [36]. Further investigation of the role of inflammatory cell infiltration in the heart during disease is thus required.

The sterile and controlled conditions of the murine study, with reduced biological variance because of single strain and sex use, provide a more uniform response with respect to analyzed remodeling. However, the development of hypertension induced by AngII is known to differ based on the sex of the animal, with growing evidence that sex hormones interfere with the renin-angiotensin system [37]. Therefore, despite a mixed human cohort with respect to sex, a limitation of this study is the use of male mice as the only comparator to the human setting, highlighting necessary future research. We also acknowledge that, based on the nature of the tertiary referral service, a limitation of this study is the reduced number of human samples available for research, along with the lack of human hypertensive heart failure samples. Furthermore, while a murine model introduces a single insult to establish disease, humans have the potential for multiple insults, thus impacting disease etiology, such as genetic variability or diet. Despite these limitations, this study showcases the clinical relevance of the murine model by mirroring human disease progression. While our study consists of a UK-based cohort only, we believe that our results provide a valuable representation of the progression of hypertensive remodeling globally. We anticipate that the optimization of murine analysis to the human setting could be a tool to assist in the post-mortem assessment of cardiac causes of death, both in the context of hypertension and expanded to include other cardiomyopathy types.

Clinically, hypertension is first treated by agents to normalize blood pressure, e.g., angiotensin-converting enzyme inhibitors (ACEi) and angiotensin receptor blockers (ARB) [38]. However, not all individuals respond to ACEis and ARBs, with beta-blockers being the next point of call, aiming to block the action of adrenaline, thereby lowering the cardiac workload and blood pressure. While beta-blockers work directly on cardiomyocytes due to the presence of beta-adrenergic receptors, treatments targeting AngII work on the whole cardiovascular system, involving peripheral control of blood pressure, tissue-specific control of fibrosis, and maladaptive responses in cardiac cells [39]. Studies have thus identified that AngII-targeting antihypertensive medications can reduce inflammation within the vasculature, whereas beta-blockers show no beneficial effects with respect to inflammation [40]. Treatment for hypertensive heart disease follows the same guidelines as hypertension, which work to reduce the cardiac workload by decreasing blood pressure but are unable to reverse established cardiac remodeling. We have identified inflammation as a key trigger for remodeling, with infiltration of inflammatory cells remaining elevated and perivascular fibrosis increasing over time. The difficulty here, however, is identifying a suitable target for anti-inflammatory treatment, as therapeutic interventions aimed at targeting cytokines have proved controversial. For example, studies demonstrated that cytokines such as TNFα and IL6 can promote cardiac remodeling with the inhibition of these molecules, or their receptors, attenuating pathological changes [35]. However, immunomodulation of TNFα (by infliximab or etanercept) worsened clinical outcomes in 2 large Phase-III trials, likely reflecting a lack of understanding of how TNFα mediates physiological vs. pathological cardiac remodelling and whether targeting at the cytokine/receptor level is most appropriate [41]. Nevertheless, by quantifying and elucidating specific remodeling changes, this study provides a framework that proposes that targeting inflammation alongside blood pressure may halt progression to HHD and ultimately cardiac failure.

## 4. Materials and Methods

### 4.1. Human Study Population 

The CRY Centre for Cardiovascular Pathology is a specialist referral centre for cases of suspected SCD across the UK, receiving ~800 cases per year and with a database of over 7000 cases. For the purposes of this study, SCD was defined as a natural death occurring from a cardiac cause, with death occurring within one hour of onset of symptoms in witnessed cases and 24 h of the individual last being seen alive when unwitnessed [25]. Pathological examination of all cases was conducted with the permission of Her Majesty’s Coroner and next-of-kin according to specific guidelines, and all relevant information regarding the deceased was provided by the referring pathologist/coroner.

Cases were identified from a cohort of 7039 referrals between 1994 and 2020. Hearts were examined by two expert cardiac pathologists. Clinically hypertensive (*n* = 9) and hypertensive heart disease (HHD; *n* = 10) cases where cardiac tissue was retained with consent for research were age and sex matched to controls with morphologically normal hearts and sudden adult death syndrome (SADS) as a cause of death (*n* = 10). Clinically hypertensive cases had a pre-mortem hypertension diagnosis by general practitioners, documented on medical records, and a morphologically normal heart upon post-mortem examination with SADS listed as the cause of death. Pathological diagnostic criteria for HHD cases included a clinical history of hypertension, increased heart weight (>500 g in males, >400 g in females) and thickened left ventricular wall (>15 mm) in the absence of other causes as previously described [25]. Microscopic criteria included myocyte hypertrophy with/without interstitial fibrosis in the left ventricle. Patient demographics and available clinical data are included in Appendix A Table A1. It is a recognised limitation of the post-mortem tertiary referral service that not all clinical information is received.

The study was approved by the London-Stanmore Research Ethics Committee (10/H0724/38).

### 4.2. Ethical Statement and Source of Animals

Procedures were performed in accordance with the European Parliament Directive 2010/63/EU on the protection of animals used for scientific purposes and the U.K Animals (Scientific Procedures) Act 1986. Wild-type C57Bl/6J mice and Alzet osmotic minipumps were from Charles River UK.

### 4.3. In Vivo Mouse Studies

Wild-type male (8–10 weeks) mice were housed at the Biological Research Facility at St. George’s University of London (UK registered with a Home Office certificate of designation). Animals were allowed to acclimatise (2 weeks) before experimentation. Mice were randomly allocated to the experimental group. Drug delivery used Alzet osmotic pumps (model 1004D), filled according to the manufacturer’s instructions. Mice received minipumps filled with vehicle (acidified PBS) or AngII (0.8 mg/kg/day; Merck; Darmstadt, Germany) to induce hypertension exactly as previously published [13,14].

Mice were euthanized at study endpoints by cervical dislocation while under anaesthesia (2% isoflurane), and death was confirmed by severing the femoral artery. Hearts were excised quickly, washed in PBS, blotted to remove excess PBS, weighed, and sliced mid-ventricle with the apex snap-frozen in liquid N_2_ and the atria/ventricles fixed in 10% buffered formalin for histology and immunohistochemistry.

### 4.4. Histology and Assessment of Myocyte Size and Fibrosis 

Histological staining and analysis in the hypertensive mouse were performed as previously described [13,14], assessing general morphology by haematoxylin and eosin (H&E) and fibrosis by picrosirius red (PSR). For assessment of myocyte size and fibrosis in human tissues, segments from the right ventricle, interventricular septum, left ventricular anterior wall and left ventricular posterior wall were taken as is standard in the CRY facility [25]. Following standard tissue processing, 5 μm sections were cut and stained for both H&E and PSR. The murine assessment method was applied to human stained sections to allow for analysis of remodelling in the human hearts, which was performed by researchers blinded to disease groups. For analysis of human perivascular fibrosis, vessels were defined by the presence of a media with a defined lumen (diameter > 100 µm).

### 4.5. Immunohistochemistry 

Deparaffinised, rehydrated human and murine sections were heated in 10 mM Tris-HCl buffer (pH 10) for antigen retrieval. Tissue sections were then permeabilised in 0.2% Triton in tris-buffered saline (TBS) and blocked by incubating in TBS containing 1% BSA and 10% goat serum. Sections were next incubated with primary antibody made up in TBS containing 1% BSA and 0.025% Triton X-100. Primary antibodies included monoclonal mouse anti-human CD45 (Dako; Glostrup, Denmark: M0701) for human tissue and monoclonal rat anti-mouse CD45 (Biolegend; London, UK: 103101) for murine tissue. For negative controls, murine myeloma IgG1 control (Sigma: M5284) was used for human staining, and rat IgG control (Vector Laboratories; Burlingame, U.S.A: 1–4000) for murine staining. Following incubation, 0.3% hydrogen peroxide in TBS was used to block endogenous peroxidase activity. Immunoperoxidase staining was performed using RTU Biotinylated anti-mouse (human tissue; Vector Laboratories: BP-9200) or anti-rat (murine tissue; Vector Laboratories: BP-9400) IgG and horseradish peroxidase avidin D-based detection (Vector laboratories: A-2704) with DAB staining (Sigma Aldrich; St. Louis, U.S.A: 11718096001). Slides were counterstained with Harris Haematoxylin and blued in lithium carbonate. Immunosignal slides were scanned using the NanoZoomer 2.0RS (Hamamatsu, Japan) and analysed using NDP-View-2 (Hamamatsu, Japan). The count of CD45+ cells was assessed by researchers blinded to human disease group or murine treatments. We acknowledge the use of the Image Resource Facility, St. George’s University of London, for immunohistochemical staining.

### 4.6. RNA Preparation and qPCR

Total RNA was prepared by homogenizing frozen murine heart tissue with 1 mL TRIzol followed by 200 μL chloroform. RNA was prepared and reverse transcribed to cDNA as previously described [42]. Reverse Quantitative PCR (qPCR) was performed as previously described [13]. See Appendix A Table A2 for primer sequences. Results were normalised to *Gapdh* as reference gene, with relative quantification using ΔCt (threshold cycle) method; relative expression calculated as 2^−ΔΔCt^ and normalised to vehicle.

### 4.7. Statistical Analysis

Data were curated in Microsoft Excel (Microsoft 365; Redmond, WA, USA) and GraphPad Prism (9.0; San Diego, CA, USA). Statistical analysis was performed using GraphPad Prism with one-way ANOVA and Holm-Sidak multiple comparison test. Graphs were plotted with GraphPad Prism.

### 4.8. Data Analysis and Availability

For welfare monitoring purposes, individuals conducting in vivo studies were not blinded to treatment conditions. Data analysis was performed by researchers blinded to intervention/treatment. All data generated or analysed during this study are available from the corresponding author.

## Data Availability

The data presented in this study are available upon reasonable request from the corresponding author.

## Appendix A

**Table A1:** Human patient demographics and clinical data.

Human Cohort	Sex (M:F)	Age at Death (Mean, S.D)	On B.P Medication	Identified Medications
Control	1:1.5	55.1 ± 20.8	N/A *	N/A *
Clinical hypertension	1:1.3	50.3 ± 12.5	44%	ACE inhibitors (Ramipril, Perindopril), beta-blocker (Bisoprolol) and calcium channel blockers (Felodipine, Amlodipine)
Hypertensive heart disease	1:1.5	55.0 ± 20.0	80%	ACE inhibitors (Ramipril, Perindopril, Lisinopril), beta-blocker (Bisoprolol), calcium channel blocker (Amlodipine) and diuretics (Bumetanide, Furosemide)

* Not applicable given control samples were normotensive.

**Table A2:** Mouse primer sequences.

Gene	Forward (Sense Primer)	Reverse (Antisense Primer)
*Gapdh*	TCACCACCATGGAGAAGGC	GCTAAGCAGTTGGTGGTGCA
*Nppa*	GATGGATTTCAAGAACCTGCTAGA	CTTCCTCAGTCTGCTCACTCA
*Nppb*	TCCAGCAGAGACCTCAAAATTC	CAGTGCGTTACAGCCCAAA
*Myh7*	GAGATCGAGGACCTGATGG	TCATACTTCTGCTTCCACTCA
*Col1a1*	TCGTGGCTTCTCTGGTCTC	CCGTTGAGTCCGTCTTTGC
*Col3a1*	GGAACCTGGTTTCTTCTCACC	TAGGACTGACCAAGGTGGCT
*Cd45*	ATGGTCCTCTGAATAAAGCCCA	TCAGCACTATTGGTAGGCTCC
*Cd14*	GGCGCTCCGAGTTGTGACT	TACCTGCTTCAGCCCAGTGA
*Il1b*	AAGGGCTGCTTCCAAACCTTTGAC	ATACTGCCTGCCTGAAGCTCTTGT
*Il6*	ATCCAGTTGCCTTCTTGGGACTGA	TAAGCCTCCGACTTGTGAAGTGGT
*Il11*	AATTCCCAGCTGACGGAGATCACA	TCTACTCGAAGCCTTGTCAGCACA
*Tnfa*	TCTCATGCACCACCATCAAGGACT	ACCACTCTCCCTTTGCAGAACTCA
